# Bioimpedance to Assess the Body Composition of High-performance Karate Athletes: Applications, Advantages and Perspectives

**DOI:** 10.2478/joeb-2021-0009

**Published:** 2021-11-20

**Authors:** Luciana Rossi

**Affiliations:** 1 Scientific Academic Department (DAC) of the São Paulo Karate Federation (FPK). Post-doctoral degree from School of Pharmaceutical Sciences – University of São Paulo (USP), São Paulo – SP, Brazil

**Keywords:** Martial arts, combat sports, body composition, anthropometry, bioimpedance

## Abstract

Karate, a millennial martial art, was recently inserted among traditional Olympic combat sports for the Olympic Games in Japan. The aim of the present study is to determine, through bioimpedance analysis, the body composition of high-performance athletes participating in the São Paulo Olympic Karate Project. Participants were 22 athletes of both sexes, average age of 23.6 (6.0) years old, body mass of 66.6 (13.5) kg and fat percentage of 16.6 (5.5)%. Bioimpedance test indicated significant differences between sexes related to sexual dysmorphia, which had an impact on bioelectrical variables. Through comparative evaluation between male and female athletes, this study contributes to body composition analysis, indicating that, in the future, related bioimpedance tests should be used beyond their classical application, including phase angle, muscle function and other attributes.

## Introduction

Karate, or “empty hand way”, is modern combat sport based on Olympic competition standards [1]. For the Olympic competition, there are two modalities: *Kumite*, a fight between two opponents that requires programing of rapid responses suitable to the defense and/or attack process, and *Kata*, a sequence of pre-determined movements in a fight against imaginary opponents [2]. Olympic combat sports (OCS) include boxing, judo, taekwondo and wrestling as sport modalities and represent 20% – 25% of the total medals competed for in the Olympics [1]; such estimation must increase with the inclusion of karate in the Games in Japan.

Classification into weight categories is required for OCS and, in the search for competitive advantages, several athletes commonly manage to fit in weight categories that are 5% – 10% lighter than their usual weight, which may increase their risk of developing harmful attitudes to their health and performance as techniques such as spitting, vomiting, steam baths, saunas and laxative or diuretic use [3,4]. Learning and monitoring changes in the body composition is essential to define and control the weight category; moreover, higher fat percentages are related to possible negative performance in locomotion and balance-breaking activities in combat sports [4,5].

Among the methods adopted to assess body composition, Bio-electrical Impedance Analysis (BIA) has been widely employed since the 1990’s for showing bio-electrical properties [6]. There is also increasing evidence of the applicability of phase angle (PhA) in sports [7]. Recent studies have strongly supported the hypothesis that PhA is a representative index of the extracellular water (ECW)/ intracellular water (ICW) ratio or body cell mass (BCM) [8,9]. Although still speculative, PhA must be further investigated for its reliability as an index to identify critical individual characteristics of performance, evaluate the effects of training programs, manage the strategies adopted in sports involving weighing, and other applications [10]. Matias *et al*. [11] found in judo athletes that PhA did not differ from during a period when body weight was stable, and prior to competition. Changes in PhA were directly associated with changes in serum and red blood cells (RBC). These results highlight that in elite athletes PhA may be an indirect indicator of muscular function.

The aim of the present study is to determine, through doubly indirect methodology, the body composition of high-performance karate athletes, the most recent Olympic combat sport, as well as to indicate new and emerging approaches to bioimpedance analysis related to both health and athletic performance.

## Materials and methods

### Sample

High-performance karate athletes (n=22) participating in the São Paulo Olympic Karate Project of São Paulo State Karate Federation (“Federação Paulista de Karate” – FPK) were evaluated. Of these, 54.5% (n=12) were men.

### Sampling site

Data were collected from an Integrated Health Center (IHS) at a university in São Paulo, Brazil.

### Method

In this cross-sectional study with a retro-analytical component, data were obtained under same conditions, at the morning after at least 8 hours of jejum, previously oriented by nutritionist. Inclusion criteria were age within the range of 19–59 years old for both sexes and euhydration. The included athletes agreed to sign a free and informed consent term before for appointment at the Integrated Health Center and not dehydrated.

### Data collection and variables

The data collected from 22 records were body mass (kg), obtained with a Filizola mechanical anthropometric scale (0.1 kg precision and 150 kg capacity), and height, measured with a stadiometer coupled to the scale (1 mm precision and 2 m capacity). For measurements, athletes wore minimal clothing and were positioned in Frankfurt plan [12]. These variables were used to calculate the body mass index (BMI: kg/m^2^).

Bioimpedance analysis was performed with Biodynamics 310e equipment at 50 KHz. Previous instructions were given to the athletes by phone call concerning the preparation for the test; then, electrodes were attached as follows: right foot, distal electrode on the base of the middle toe and proximal electrode between the medial and lateral malleoli, and right hand, distal electrode on the base of the middle finger and proximal electrode coinciding with the styloid process. The distance between electrodes was kept over 5 cm and patients were in dorsal decubitus with their right foot and hand slightly separated from their trunk [1,9]. From such evaluation, the following bioelectrical variables were obtained: resistance (R: Ω), reactance (Xc: Ω) and phase angle (PhA: degrees); the latter was calculated by the equation arctan [Xc (reactance)/R (resistance)] x 180º/π [7]. The following data were also measured: fat percentage (%F), fat mass (FM: kg), fat-free mass (FFM: kg), and total body water (TBW: litters).

The muscle mass hydration percentage was also found and, according to the manufacturer’s instructions, BIA results are reliable provided that such percentage is within the euhydration limits (69.0% and 75.0%); thus, 7 athletes who were hypohydrated were excluded, remaining n=22.

### Statistical analysis

Continuous variables were descriptively analyzed by central tendency (mean) and dispersion (standard deviation) values. To detect statistical differences between male and female athletes, Wilcoxon’s non-parametric test was applied at 5% significance level to reject the Null Hypothesis (H0: μ = 0). R Core Team [13] was adopted for the statistical analyses.

### Informed consent

Informed consent was obtained from all individuals included in this study.

### Ethical approval

The research related to human use was complied with all relevant national regulations, institutional policies and in accordance with the tenets of the Helsinki Declaration, and was approved by the authors’ institutional review board under the code number CAAE: 12867319.7.0000.5492.

## Results

The means obtained for high-performance karate athletes participating in São Paulo Olympic Karate Project of FPK were 23.6 (6.0) years old; BMI of 23.38 (2.77) kg/m^2^, and fat percentage of 16.6 (5.5)%; thus, they were classified as eutrophic, according to WHO [14], and their body fat level was below the average for physically active individuals [12].

Complementary anthropometric profile of athletes in general and according to sex is shown in Table 1.

**Table 1:** Data from the anthropometric assessment of high-performance karate athletes.

	Mean (standard deviation)			
*Variable*	General n=22	Men n=12	Women n=10	*p*
*Age (years)*	23.6 (6.0)	23.8 (6.9)	23.4 (4.9)	0.435
*Weight (kg)*	66.6 (13.5)	75.6 (8.8) ^#^	55.8 (9.7)	< 10^-5^
*Height (m)*	1.68 (0.10)	1.75 (0.04) ^#^	1.59 (0.08)	< 10^-6^
*BMI (kg.m^-2^)*	23.38 (2.77)	24.51 (2.33) ^#^	22.02 (2.74)	0.016
*Resistance (Ω)*	489.1 (84.4)	436.7 (39.6) ^#^	552.0 (81.6)	0.0002
*Reactance (Ω)*	63.0 (9.4)	58.6 (6.6) ^#^	68.4 (9.8)	0.0055
*PA (degrees)*	7.4 (0.6)	7.6 (0.5) ^#^	7.1 (0.6)	0.014
*FM (%)*	16.6 (5.5)	13.0 (3.9) ^#^	21.0 (3.6)	< 10^-5^
				
*FM (kg) FFM (kg)*	10.8 (3.5) 55.8 (12.9)	10.0 (3.9) 65.6 (6.3) ^#^	11.8 (3.0) 44.1 (7.5)	0.123 < 10^-7^
*TBW (L)*	39.7 (9.5)	46.9 (5.1) ^#^	31.1 (5.1)	< 10^-7^
*%WP*	71.1 (1.4)	71.4 (1.6)	70.7 (1.1)	0.128

ANOVA: analysis of variance; BMI: body mass index; PhA: phase angle; FM:fat mass; FFM: fat-free mass; TBW: total body water; BMR: basal metabolic rate; %WP: water percentage in fat-free mass. #Statistically significant difference between men and women.

**Figure 1:**
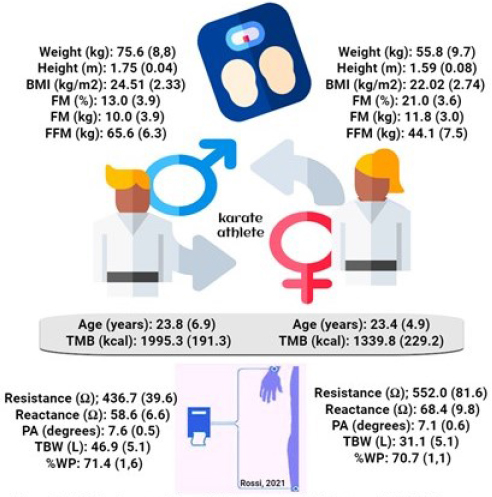
BMI: body mass index; PA:phase angle; FM: fat mass; FFM: fat-free mass; TBW: total body water; BMR: basal metabolic rate; %WP: water percentage in fat free mass.

For the studied sample of karate athletes, there were statistically significant differences between sexes for the anthropometric variables (Figure 1), except age, fat mass (kg) and water percentage in fat-free mass (Table 1).

**Figure 2:**
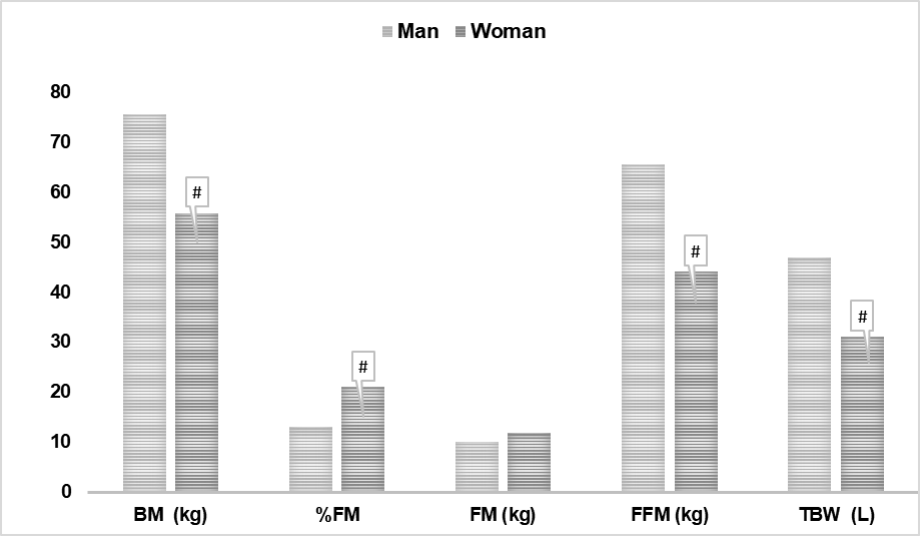
Major components of the body composition assessment for high-performance karate athletes through bioimpedance (fat-free mass, fat mass and total body water) in the sample and between sexes. BM (body mass); %F (fat percentage); FM (fat mass); FFM (fat-free mass) and TBW (total body water). ^#^statistically different between sexes.

As regards the evaluation of body composition based on three components, the major significant differences in the sample and between sexes are evidenced in Figure 2. All were statistically different between sexes, except for fat mass.

## Discussion

Anthropometric and body composition variables indicated eutrophy pattern (body mass, height and BMI) between sexes. However, the known sexual dysmorphic pattern was maintained since men showed higher weight, height, BMI, fat-free mass and total body water, except fat mass, while women had higher values of the bioelectrical variables resistance and reactance, except phase angle. Similar results were obtained by Marini *et al*. [8] for 202 athletes of different sports (athletics, basketball, handball, judo, karate, pentathlon, rugby, soccer, swimming, triathlon, and volleyball). Total body water percentage, determinant of the hydration status of the sample, did not differ between sexes.

For OCS, in which athletes compete in well-defined weight categories, eventual changes in their body composition, due to higher weight from fat accumulation, may negatively impact their athletic performance or even lead them to compete in a heavier category, drastically reducing their performance [15]. Rapid and accurate routine evaluation of the body fat percentage for athletes is essential, as well as methods that allow its monitoring. In an investigation of the anthropometric profile of elite karate athletes, a wide variation in fat percentage for men (7.5 to 18.6%) was found, while for women it was 18.6 (3.2)% [15]. In addition, a recent study conducted with 24 high-performance karate athletes recorded fat percentage of 18.6 (4.0)% [16].

Bioimpedance has the additional advantage of determining a parameter related to the hydration status based on TBW values, which constitutes an analysis of the body composition by three components, differing thus from the anthropometric method, which determines only two body components [17] (Figure 1). Changes in total body water, intentional or not, must be monitored since modalities involving body weight control require attention and surveillance by the multidisciplinary team regarding the use of harmful methods to sharply reduce the body mass [18,19]. Although weight in methods have been widely used by fighters due to their efficacy in drastically reducing weight, these methods employ dehydration in particular, which may negatively impact the general performance depending on the loss magnitude, the method (e.g., active vs passive dehydration) and the origin of water loss [20]. Lower aerobic resistance due to dehydration is well documented; however, data on strength and potency, skills of greater relevance in combat sports and karate itself, are less evident and poorly investigated [19,21]. The detectable negative effects on motor skills, cognitive performance and movement patterns specific to combat sports progressively manifest after ≥2% loss in total body mass [19,21,22].

Recently, Marini and collaborators [8] investigated the relationship between the body composition of athletes of diverse sport modalities, including OCS (judo and karate), and the phase angle; they reported values close to those found for the karate athletes in the present study, also statistically differing between men and women: PhA was 7.7 (0.8) degrees for men (n=139) and 6.8 (0.8) degrees for women (n=63).

Considering the phase angle value, its practical application must go beyond classical studies that use it as an index of the ratio between intra and extracellular water, or body cell mass and cell integrity [7,11]; there are also different approaches, as is the case for judo, in which the phase angle is related to magnesium status and can be considered an indirect indicator of the muscle function, which is directly related to health status and consequently athletic performance [10].

## Conclusion

This study aimed to record the body composition of high-performance karate athletes of both sexes through doubly indirect methodology. Bio-electrical Impedance Analysis for three components (fat-free mass, fat mass and total body water) was adopted given the importance of evaluating body changes, desirable or not, due to alterations in the hydration status. The differences found between men and women were consistent with studies involving OCS athletes, contributing thus to better understanding the application of bioimpedance in the follow-up or maintenance of the body composition, during training and competitions for this modality recently incorporated in the Olympic Games.
